# Prevalence of Serum Celiac Antibodies in a Multiracial Asian Population-A First Study in the Young Asian Adult Population of Malaysia

**DOI:** 10.1371/journal.pone.0121908

**Published:** 2015-03-23

**Authors:** Theresa Wan-Chen Yap, Weng-Kai Chan, Alex Hwong-Ruey Leow, Ahmad Najib Azmi, Mun-Fai Loke, Jamuna Vadivelu, Khean-Lee Goh

**Affiliations:** 1 Department of Medical Microbiology, Faculty of Medicine, University of Malaya, 50603 Kuala Lumpur, Malaysia; 2 Department of Medicine, Faculty of Medicine, University of Malaya, 50603 Kuala Lumpur, Malaysia; Tulane University, UNITED STATES

## Abstract

**Background:**

Celiac disease (CD) is an immune-mediated disorder induced by the ingestion of gluten in genetically susceptible persons. The prevalence of CD in Malaysia is unknown. We aim to determine the seroprevalence of CD antibodies and also investigate the correlation between *H*. *pylori* infection and CD in the young and healthy multiracial Malaysian population.

**Methods:**

Healthy young adult volunteers between the ages of 18–30 years were consecutively recruited from June 2012 to May 2014 at the University of Malaya Medical Centre (UMMC), Kuala Lumpur. Serum samples from all the participants were tested for anti-gliadin antibody immunoglobulin A/immunoglobulin G (IgA/IgG) and anti-tissue transglutaminase antibody (tTG) IgA/IgG. Samples positive for both anti-gliadin and anti-tTG were further validated for anti-human endomysial IgA antibodies (EmA). Serological diagnosis of CD was made when anti-gliadin, anti-tTG and anti-EmA were positive.

**Results:**

562 qualified participants with mean age 24 ± 2.4 years old were recruited into our study. CD was found in 7 participants where most of them were asymptomatic and unaware of their CD status. The median of anti-gliadin and anti-tTG IgA/IgG value was 38.2 U/ml (interquartile range, 28.3–60.4 U/ml) and 49.2 U/ml (interquartile range, 41.1–65.9 U/ml), respectively. Seroprevalence of CD antibodies was 1.9% (6 out of 324) in female while only 0.4% (1 out of 238) in male. Seroprevalence among Malay was 0.8% (2 of 236), Chinese was 1.7% (3 of 177) and Indian was 1.3% (2 of 149). Overall, seroprevalence of CD antibodies in healthy asymptomatic adults in the Malaysian population was 1.25% (95% CI, 0.78%-1.72%). No significant relationship was discovered between CD and *H*. *pylori* infection.

**Conclusions:**

The seroprevalence of CD antibodies in healthy young adults in the Malaysian population was 1.25% (1 in 100). CD is underdiagnosed and it could be a much greater problem in Malaysia than previously thought.

## Introduction

Celiac disease (CD) is an immune-mediated enteropathy induced in genetically susceptible individuals by the ingestion of gluten or related rye and barley proteins [1]. The classic clinical manifestations for CD are mostly gastrointestinal in nature such as diarrhea, malnutrition, weight loss, steatorrhea and edema secondary to hypoalbuminemia [2]. Subsequently, the disease has also now been diagnosed in patients suffering from a variety of atypical symptoms such as anemia or osteoporosis and even in asymptomatic subjects (also formerly known as silent celiac disease or latent celiac disease) [2–4]. CD is a lifelong disorder that affects both genders of all ages and is one of a few immune disorders that can be controlled when diagnosed and treated early [5].

The diagnostic gold standard of celiac disease in current clinical practice is a combination of invasive small intestinal biopsy and the concomitant presence of a positive celiac disease–specific serology [2]. Nonetheless, advances in diagnostic testing for CD now allow accurate disease prevalence estimates in large populations by using noninvasive serologic testing alone [6].

Previously, this disease was considered to be uncommon and the prevalence was estimated to be as low was 0.03% globally [7]. With the advent of highly sensitive and specific serological tests including anti-tissue transglutaminase (tTG) antibody, anti-gliadin (AGA) antibody and anti-endomysial (EmA) antibody assays, increased incidences of CD have been reported [3, 8]. Large-scale screening studies using these assays in different populations have demonstrated that the frequency of the disease is much higher than previously thought. Recent studies have shown that the prevalence of CD in the United Kingdom and the United States has increased to approximately 1% [9] and 0.71% [10] respectively.

There has however been a paucity of studies on CD from the Asia-Pacific region [11, 12]. CD was always thought to be rare in East Asia but several studies from China and Japan have shown that this disease could have been underdiagnosed [13–17]. A recent study from northern India showed a prevalence of 1.04% which is more common than previously recognized in India [3]. Reports from New Zealand and Australia with a predominant Caucasian population understandably shown a relatively high prevalence of 1.2% [18] and 0.4% [19] respectively. In the Middle East, prevalence rates range from 0.6%-1.2% was reported in Iran, Israel, Syria and Turkey [20–25]. There have however been no clinical reports of CD from Southeast Asia (including Malaysia) [12].

The disappearance of *Helicobacter pylori* has been correlated with an increase in several diseases including allergy [26, 27], asthma [26, 28] and gastroesophageal reflux disease (GERD) [29]. It is believed to be related to immunological changes with the eradication of *H*. *pylori* in stomach. Interestingly too, an inverse relationship between CD and *H*. *pylori* infection has been reported [30]. This is a new observation which needs to be explored further.

This study is the first clinical report of the seroprevalence of CD antibodies in the Malaysian population. The primary aim of this study is to determine the prevalence of CD based on serological assay of CD antibodies in the young multiracial Malaysian population. We also aimed to investigate the correlation between *H*. *pylori* infection and CD in our study population.

## Methods

### Subjects and Study population

Our study was part of a cohort study named HEALTHY Study conducted at the University of Malaya Medical Centre (UMMC) to screen for the prevalence of celiac disease and *H*. *pylori* infection among healthy young Malaysians. Volunteers for this study were consecutively recruited from June 2012 to May 2014 on the University of Malaya and MAHSA University campus. The recruited volunteers were healthy young Malaysian adults with an age range from 18 to 30 and consisted of predominantly university students. The exclusion criteria for the study were diabetes, hyper or hypothyroidism, prior gastric or bariatric surgery, prior documented treatment of *H*. *pylori*, antibiotic use within 4 weeks of enrollment, steroid or other immunomodulating drug use within 4 weeks of enrollment and Charlson weighed comorbidity index 17 ≤2. The study protocol was reviewed and approved by Medical Ethics Committee, UMMC (Ref No. 877.1; Date of Approval: September 21^st^, 2011). Written informed consent was obtained from volunteers prior to study participation. Volunteers also filled up a questionnaire on the clinical history/gastrointestinal symptoms. Symptoms of diarrhea, bloating and constipation were defined based on the Rome III criteria [31].

Serum was collected from the study cohort of 569 subjects. Seven subjects were excluded either because of insufficient volume of serum or the blood samples were badly hemolyzed at collection. All the sera were stored at −80°C freezer until analyzed.

### Study protocol

Sequential serologic testing, with anti-gliadin antibodies (AGA) immunoglobulin A/immunoglobulin G (IgA/IgG) and anti-tissue transglutaminase antibodies (tTG) IgA/IgG used as screening tests and anti-endomysial IgA antibodies (EmA) used as a confirmatory test for persons with positive anti-AGA and anti-tTGA, were used to screen for CD in young adult population in Malaysia.

### Measurement of gliadin antibodies

Sera were analyzed for anti-human gliadin antibodies (AGA) IgA/IgG by enzyme-linked immunosorbent asaay (ELISA) using AESKULISA GliadinCheck ELISA kit (AESKU Diagnostics, Wendelsheim, Germany). Result interpretation was as follow: normal, < 24 U/ml; positive, > 24 U/ml.

### Measurement of tissue transglutaminase antibodies

Sera were analyzed for anti-human tissue transglutaminase (tTG) IgA/IgG by enzyme-linked immunosorbent asaay (ELISA) using AESKULISA CeliCheck ELISA kit (AESKU Diagnostics, Wendelsheim, Germany). Result interpretation was as follow: normal, < 24 U/ml; positive, > 24 U/ml. This test has been validated previously with high sensitivity, specificity and accuracy [32, 33].

Samples with positive for both anti-AGA and anti-tTG results (titers >24 U/ml) were further validated for anti-human endomysial IgA antibodies (EmA) by indirect immunofluorescence assay (IFA).

### Measurement of endomysial antibodies

Anti-human endomysial IgA antibodies (EmA) in serum were determined using monkey esophagus as substrate and visualized by indirect immunofluorescence (GA Generic Assays GmbH, Dahlewitz, Germany). Subject sera were serial diluted to 1:10 and 1:40. A serum dilution is considered positive for anti-EmA if the fluorescent staining is at an intensity of 1+ or greater with a clearly discernable pattern of fluorescence in the musclaris mucosae (staining of the endomysium around the smooth muscle fibers). A positive fluorescence at dilutions equal to or greater than 1:10 was considered positive.

### Measurement of anti-*Helicobacter pylori* antibodies

Anti-*H*. *pylori* IgA and IgG in the serum were determined by ELISA using Pyloriset EIA-A III & EIA-G III (Orion Diagnostica, Espoo, Finland) according to manufacturer’s instruction. These two tests have been previously validated and demonstrated a high sensitivity and specificity [34, 35]. For purposes of this study, a value of ≥ 20 U/ml was regarded as a positive test for *H*. *pylori* while a result of < 20 U/ml was regarded as a negative test for *H*. *pylori*.

### Statistical analysis

Data were presented as mean ± standard deviation, median (with 25%-75% interquartile range), or n (%). Seroprevalence of CD antibodies in the Malaysian population was measured using population proportion and 95% confidence intervals (CI). Pearson’s chi-square test or Fisher’s exact test where appropriate and followed by logistic regression were used to determine any association between demographics factors (gender, ethnicity), clinical history, presence of gastrointestinal symptoms and anti-*H*. *pylori* antibodies to CD serology. Multivariate regression analysis was also performed adjusting for independent variables. All statistical calculations were performed using SPSS software version 20.0 (SPSS Inc., Chicago, IL); a two-tailed p-value of < 0.05 was considered significant.

## Results

**Fig 1:**
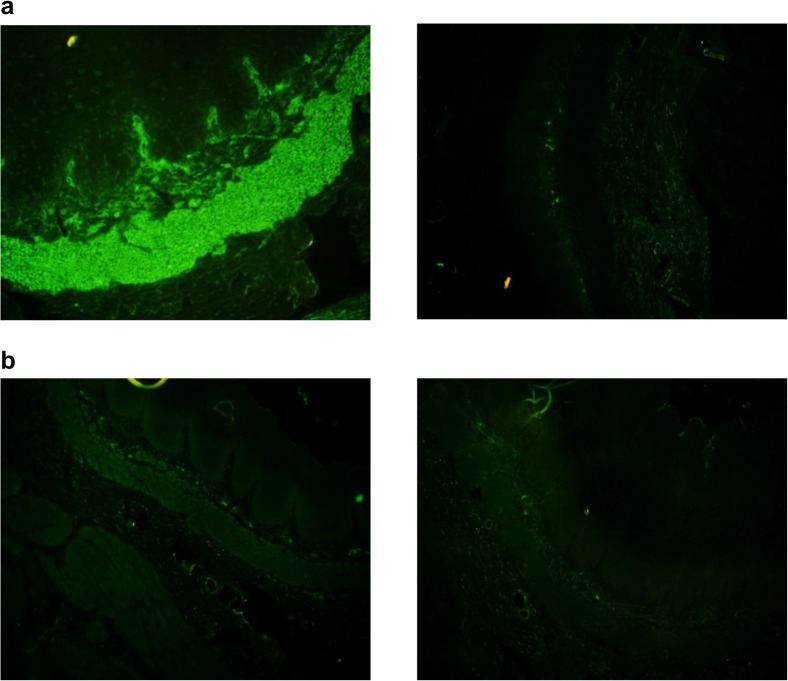
Fluorescence images of detection anti-EmA antibodies using indirect immunofluorescence assay. (A) Image on the left is positive control; image on the right is negative control under 100X magnification. (B) An example of anti-EmA positive sample at 1:10 dilution (left image) and 1:40 dilution (right image) under 100X magnification. Fluorescent staining with intensity of 1+ or greater with a clearly discernable pattern of fluorescence in the musclaris mucosae (staining of the endomycium around the smooth muscle fibers) was observed.

There were 562 qualified participants with mean age 24 ± 2.4 years old and mean BMI 21.8 ± 5.7 kg/m^2^ were recruited into our study. 42.3% (238) of the participants were male and 57.7% (324) were female where they comprised of 42%, 31.5% and 26.5% of Malay, Chinese and Indian respectively (Table 1). Anti-gliadin IgA/IgG results were positive in 36 (6.4%) and negative in 526 (93.6%) whilst Anti-tTG IgA/IgG results were positive in 16 (2.8%) and negative in 546 (97.2%). Eight of them with both anti-gliadin and anti-tTG positive results (titers >24 U/ml) were further validated for anti-human EmA antibodies by IFA. Of the 8 anti-gliadin and anti-tTG positive samples, only 7 (1.25%) were considered to have serologically diagnosed CD with positive fluorescence at dilutions equal to or greater than 1:10. Two (0.4%) serology positive subjects also showed higher anti-human EmA titer with positive fluorescence detected at dilutions equal to or greater than 1:40 (Fig. 1 and Table 1).

**Table 1:** Demographics characteristics and serological results of the study population.

Characteristic	Total (n = 562)
**Age, years ± SD**	24 ± 2.4
**Gender, n (%)**	
Male	238 (42.3)
Female	324 (57.7)
**Ethnicity/Race, n (%)**	
Malay	236 (42.0)
Chinese	177 (31.5)
India	149 (26.5)
**Body Mass Index, kg/m** ^**2**^ **± SD**	21.8 ± 5.7
**Celiac serology**	
Positive IgA/IgG AGA	36 (6.4)
Positive IgA/IgG tTG	16 (2.8)
EmA IgA	
1:10	7 (1.25)
1:40	2 (0.40)
**Anti-*H*. *pylori* antibodies serology**	
Positive IgA	119 (21.2)
Positive IgG	39 (6.9)
Positive IgA & IgG	57 (10.1)

For these 7 serologically diagnosed CD subjects, the median of anti-gliadin and anti-tTG IgA/IgG value was 38.2 U/ml (interquartile range, 28.3–60.4 U/ml) and 49.2 U/ml (interquartile range, 41.1–65.9 U/ml), respectively. Seroprevalence of CD antibodies was 1.9% (6 out of 324) in female while only 0.4% (1 out of 238) in male. Seroprevalence among Malay was 0.8% (2 of 236), Chinese was 1.7% (3 of 177) and Indian was 1.3% (2 of 149). Overall, seroprevalence of CD antibodies in the healthy asymptomatic adults in Malaysian population was 1.25% (95% CI, 0.78%-1.72%). To determine an association between gender or ethnic groups and CD serology, Pearson’s chi-square test followed by multinomial logistic regression was performed. This analysis was also adjusted for gender (CD was reported more often diagnosed in female [36, 37]) and ethnic groups by doing a multivariate logistic regression. However, no significant relationship was found between them (data is not shown).

Furthermore, our findings also showed that there was no significant relationship between most of the clinical history/gastrointestinal symptoms and CD serology except chronic fatigue (p = 0.000) (Table 2 & Table 3). Considering a role of microbial exposure in affecting CD-risk, association between *H*. *pylori* infection and CD was also tested. Of 562 participants, 21.2%, 6.9% and 10.1% of them were tested positive for anti-*H*. *pylori* IgA, anti-*H*. *pylori* IgG and both anti-*H*. *pylori* IgA and IgG antibodies, respectively (Table 1). Of the 7 serologically diagnosed CD subjects, 71.4% of them were tested positive for anti-*H*. *pylori* IgG and/or IgA antibodies. Conversely, only 37.8% of CD negative subjects were tested positive for anti-*H*. *pylori* IgG and/or IgA antibodies. However, no significant relationship (p = 0.069) was discovered between CD and *H*. *pylori* infection (Table 4).

**Table 2:** Association between clinical history, concomitant medical illness and Celiac Disease (n* = 527).

Clinical history	CD Serology		p-value^c^
	Positive^a^, n (%)	Negative^b^, n (%)	
**Diagnosed/suspected CD**			
Yes	0 (0.0)	2 (0.4)	0.879
No	6 (100.0)	519 (99.6)	
**Family history of CD**			
Yes	0 (0.0)	2 (0.4)	0.879
No	6 (100.0)	519 (99.6)	
**Anaemia/Iron Deficiency Anaemia**			
Yes	0 (0.0)	15 (2.9)	0.673
No	6 (100.0)	506 (97.1)	
**Type 1 Diabetes Mellitus**			
Yes	0 (0.0)	1 (0.2)	0.914
No	6 (100.0)	520 (99.8)	
**Thyroid disorders**			
Yes	0 (0.0)	6 (1.2)	0.791
No	6 (100.0)	515 (98.8)	
**History of underlying malignancy**			
Yes	0 (0.0)	2 (0.4)	0.879
No	6 (100.0)	519 (99.6)	
**Hepatic diseases**			
Yes	0 (0.0)	3 (0.6)	0.852
No	6 (100.0)	518 (99.4)	

*A total of thirty-five questionnaires were missing and excluded from the statistical analysis

^a^One questionnaire was missing and excluded from the statistical analysis

^b^Thirty-four questionnaires were missing and excluded from the statistical analysis

^c^Pearson’s chi-square test was performed between clinical history and CD serology

**Table 3:** Association between gastrointestinal symptoms and Celiac Disease (n* = 527).

Gastrointestinal Symptom	CD Serology		p-value^c^
	Positive^a^, n (%)	Negative^b^, n (%)	
**Unexplained chronic diarrhea**			
Yes	0 (0.0)	28 (5.4)	0.560
No	6 (100.0)	493 (94.6)	
**Chronic Constipation**			
Yes	0 (0.0)	32 (6.1)	0.531
No	6 (100.0)	489 (93.9)	
**Bloating episodes**			
Yes	1 (16.7)	119 (22.8)	0.720
No	5 (83.3)	402 (77.2)	
**Unexplained weight loss**			
Yes	1 (16.7)	17 (3.3)	0.072
No	5 (83.3)	504 (96.7)	
**Chronic fatigue**			
Yes	1 (16.7)	5 (1.0)	0.000
No	5 (83.3)	516 (99.0)	

*A total of thirty-five questionnaires were missing and excluded from the statistical analysis

^a^One questionnaire was missing and excluded from the statistical analysis

^b^Thirty-four questionnaires were missing and excluded from the statistical analysis

^c^Pearson’s chi-square test was performed between gastrointestinal symptoms and CD serology

**Table 4:** Association between Celiac Disease and *Helicobacter pylori* infection.

*H. pylori*	CD Serology, n (%)		p-value^c^
Serology, n (%)	Positive	Negative	
**Total Positive**	**5 (71.4)**	**210 (37.8)**	0.069
IgG Positive	1 (14.3)	38 (6.8)	
IgA Positive	3 (42.9)	116 (20.9)	
IgG & IgA Positive	1 (14.3)	56 (10.1)	
**Negative**	**2 (28.6)**	**345 (62.2)**	
	**7 (100)**	**555 (100)**	

^c^Pearson’s chi-square test was performed between *H*. *pylori* serology and CD serology

## Discussion

This study is the first report of the prevalence of celiac-specific antibodies among healthy young adults in Malaysian population. The target population in the present study is healthy young adults encompassing three main ethnic groups with a mean age of 24 ± 2.4 years. As CD has been thought to be rare or underdiagnosed in the Asia Pacific region, the healthy young adult population is a good model to investigate the prevalence of CD. Non-invasive, highly sensitive and specific sequential serological tests were able to detect CD in healthy Malaysian youths who had not previously been diagnosed and the phenotype of CD may not be manifested yet.

Eight subjects were found to be both anti-AGA and anti-tTG IgA/IgG positive. Of the 8 subjects, only 7 of them were shown to be positive with anti-human EmA antibodies and considered to have serologically diagnosed CD. Previous studies have shown that double-positive serology is predictive of small-bowel abnormalities indicative of CD [8, 10, 38, 39]. This assumption is supported by previous studies and observations that all anti-EmA positive subjects who underwent intestinal biopsy had a lesion consistent with CD [39, 40]. As for serological examination, the world wide seroprevalence was 0.3–3.2% [10, 38, 41]. In our current study, the seroprevalence of CD antibodies in healthy young adults in Malaysian population was 1.25% (95% CI, 0.78%-1.72%), which is consistent with the previous reports. However, the true prevalence of CD in healthy young adults in Malaysian population is likely to be lower than 1.25% if histology of small-bowel biopsy results were included.

Interestingly in our study 5% of the subjects (28 out of 562) were shown to be positive for anti-AGA antibodies only but not anti-tTG antibodies. This could be suggestive of non-celiac gluten sensitivity (NCGS) [42], which is linked to the consumption of wheat and other gluten-containing food. Currently, there is no biomarker available for diagnosing NCGS in which the diagnosis can be made only by excluding celiac disease and wheat allergy [43].

Of the 562 participants, 1.9% of female and 0.4% of male were found to have serologically diagnosed CD. Even though the preponderance of female can be expected in clinically diagnosed CD cases (female to male ratio, 2:1) [36, 37], this was not the case for our serologically diagnosed CD. Univariate and multivariate logistic regression analysis showed that both male and female carried equal risk in developing CD (data not shown).

Malaysia has a unique multiracial Asian population consisting of three major Asian ethnic groups: Malay, Chinese and Indian. Racial differences have been observed in the prevalence of different diseases [44, 45] and hence association between ethnic groups and CD serology was also examined. As opposed to a recent study in the United States [10], our findings indicate that the prevalence of CD in Malaysian population did not vary with race/ethnicity. It has been suggested that genetic predisposition of the two major haplotypes, HLA-DQ2 and HLA-DQ8 play a major role in the development of CD [46]. However, HLA haplotyping was unable to be performed in our study to verify the effect of genetic risk in CD manifestation.

Another finding with particular interest was the high seroprevalence of CD antibodies found in our subjects who did not present clinical history/gastrointestinal symptoms related to CD manifestation except chronic fatigue. Such a finding was suggested to be an indicative of latent CD or potential CD [47, 48]. Although the p-value for chronic fatigue was shown to be significant, our CD-positive samples were not enough to draw a conclusive correlation between them. However, clinicians should be aware and pay further attention on this observation in CD investigations in the future in studies involving larger number of CD positive cases. It has been reported that approximately 33% of patients with potential CD were shown to develop villous atrophy during a three-year observation period [49]. Studies have demonstrated that the presence of serum tissue transglutaminase and endomysial antibodies in subjects with initially normal villous architecture on small-bowel biopsy who consumed normal amounts of gluten can predict subsequent mucosal deterioration and development of CD [49–52]. Moreover, a published survey of 1612 patients with CD in the United States revealed that the average gap between the onset of symptoms and the time of CD diagnosis was 11 years [53].

Various studies have shown that CD and gluten sensitivity have a higher prevalence of functional gastrointestinal symptoms based on Rome III criteria in both adult and children population [54–56]. The symptoms of irritable bowel syndrome (IBS) can even reach 4 times higher among children with CD in comparison with general pediatric population [57]. Although our study failed to demonstrate the association between functional gastrointestinal symptoms and CD, the result needs to be interpreted with caution due to small sample size and in addition the objective of this study that was not designed to observe association.

The true prevalence of CD of the Malaysian population as a whole may be different from what is reported in this study, which focuses only on healthy young adults. Nonetheless our study has shown that CD is underdiagnosed and it could be a much greater problem in Malaysia than has previously been appreciated. This finding is consistent with prior studies in other populations and geographic settings [3, 8, 15, 58].

A few possible explanations as to why CD is underdiagnosed globally. Primarily, most of the clinicians believe that CD is rare and their failure to acknowledge that many individuals with CD initially presents with no gastrointestinal symptoms leads to the non-performance of CD testing [8]. In addition, missed diagnosed could also result from the use of only the more widely known but less sensitive and less specific anti-AGA serological test instead of anti-EmA and anti-tTG tests [32, 59, 60]. Furthermore, endoscopists do not always obtain small-bowel biopsy specimens that could demonstrate the presence of CD even when gastrointestinal symptoms are present and a gastrointestinal endoscopy is performed [8]. Finally, failure of pathologists to recognize early features of CD (Marsh stages 1, 2 and 3a [61]) could also contribute to underdiagnosis of CD as well [8].

The prevalence of CD has risen in recent decades and a number of hypotheses have been proposed. One of the theories, the “hygiene hypothesis”, theorizes that decreased exposure to bacterial antigens may trigger autoimmunity [30]. Microbial exposure in the stomach might play a role in affecting the risk of CD. Increased CD prevalence in the United States coincides temporally with *H*. *pylori* prevalence [62]. There are studies suggesting that an inverse relationship between CD and *H*. *pylori* infection may be present. CD is triggered by the ingestion of gluten in which its digestion may be based on the pH and condition of gastric mucosa [30]. Hence, *H*. *pylori* infection might affect ingested gluten through its modification of the gastric pH or through its proteases and hence reducing its immunogenicity [63]. Alternatively, persons with *H*. *pylori* infection were suggested to recruit more gastric T-regulatory cells [64, 65] and in turn it may be more likely to up-regulate immune response to gluten [65]. However, previous studies that have investigated the relationship between these two entities have yielded conflicting results [30, 66–72]. In our study, we found no relationship between the serology of *H*. *pylori* infection and CD serology. This is probably due to the small sample size of serologically diagnosed CD which is not sufficiently significant to perform a meaningful correlation between the *H*. *pylori* infection and CD. In addition, the change of diet from traditional rice-based staple food to western wheat-based diet could be another possible reason of the significant rise of CD prevalence in Asian population like Malaysia.

The major strength of our study was the utilization of highly sensitive and specific serological tests to a healthy adult population-based sample in Malaysia. A review paper which has compared various methods for measuring autoantibodies related to CD reported that anti-tTG antibodies could be used as a reliable test for screening in the general population or in at-risk groups [73]. CeliCheck neo-epitopes (human recombinant tTG cross-linked with gliadin specific peptides) assay has also been shown to have 100% sensitivity, 96.2% specificity, and 98.1% accuracy [33]. The anti-EmA antibodies test, on the other hand, has proved to be less sensitive than the anti-tTG antibodies test but completely specific [74–77]. The positive findings in the anti-tTG antibodies assay in our study were therefore further confirmed by the anti-human EmA antibodies test to increase the accuracy of detection of CD in healthy adult population in Malaysia. Healthy adults are the preferred target population for the first and preliminary prevalence study of CD in the Malaysian population as it has been underdiagnosed in our region. Although none of the participants reported clinical diagnosis of CD, the seroprevalence of CD antibodies in healthy young adults in Malaysian population was unexpectedly high. As suggested earlier, serological assays are reliable and simple means of screening population for clinically silent CD before symptoms or signs of chronic malabsorption develop [10, 38].

This study also has several limitations. It is possible that the estimation of prevalence of CD in our population is not completely true and it might not represent the nationwide Malaysian population. The cohort size is too small to provide stable estimation for the prevalence among demographic subgroups such as gender, age and ethnic groups and also to allow determination of the health impact of undiagnosed CD such as, anemia and osteoporosis. Moreover, we were unable to confirm the diagnosis of CD histologically although the gold standard for CD is small-bowel biopsy. The participants are healthy individuals without gastrointestinal symptoms. It would not be feasible to obtain biopsies from them and hence, only less invasive serological tests were performed. Additional large-scale studies that use random sampling and include proportional representation of ethnical groups in Malaysia are warranted to confirm our findings.

In conclusion, we have undertaken the first CD epidemiology screening study in Malaysia. Sequential serologic testing offers an objective means of detecting CD early especially when it is clinically silent. Our results have shown that the seroprevalence of CD antibodies in healthy young adults in Malaysian population was 1.25% (1 in 100). We believe that CD is underdiagnosed and it could be a much greater problem in Malaysia than previously thought.
